# Beneficial Effects of Betaine: A Comprehensive Review

**DOI:** 10.3390/biology10060456

**Published:** 2021-05-22

**Authors:** Madan Kumar Arumugam, Matthew C. Paal, Terrence M. Donohue, Murali Ganesan, Natalia A. Osna, Kusum K. Kharbanda

**Affiliations:** 1 Research Service, Veterans Affairs Nebraska-Western Iowa Health Care System, Omaha, NE 68105, USA; madankumar.arumugam@unmc.edu (MK.A.); mpaal@unmc.edu (M.C.P.); tdonohue@unmc.edu (T.M.D.J.); murali.ganesan@unmc.edu (M.G.); nosna@unmc.edu (N.A.O.); 2 Department of Internal Medicine, University of Nebraska Medical Center, Omaha, NE 68198, USA; 3 Department of Biochemistry & Molecular Biology, University of Nebraska Medical Center, Omaha, NE 68198, USA

**Keywords:** alcohol, adipose tissue, betaine, diet, hepatic steatosis, methylation, S-adenosylhomocysteine, S-adenosylmethionine

## Abstract

**Simple Summary:**

A large number of studies report that medicinal herbs and many food ingredients protect against the development of liver disease because they possess antioxidant, anti-inflammatory, or anti-necrotic activities. This review focuses on the biological and beneficial effects of dietary betaine (trimethylglycine), a naturally occurring and crucial methyl donor, that restores methionine homeostasis in cells. We describe recent studies on betaine’s mechanism(s) of action as a therapeutic agent for improving indices of alcohol-induced and metabolic- associated liver disease. Due to its low cost, high tolerability, and efficacy, we suggest betaine as a promising therapeutic for clinical use to treat these aforementioned diseases as well as other liver-/non-liver-related diseases and conditions.

**Abstract:**

Medicinal herbs and many food ingredients possess favorable biological properties that contribute to their therapeutic activities. One such natural product is betaine, a stable, nontoxic natural substance that is present in animals, plants, and microorganisms. Betaine is also endogenously synthesized through the metabolism of choline or exogenously consumed through dietary intake. Betaine mainly functions as (i) an osmolyte and (ii) a methyl-group donor. This review describes the major physiological effects of betaine in whole-body health and its ability to protect against both liver- as well as non-liver-related diseases and conditions. Betaine’s role in preventing/attenuating both alcohol-induced and metabolic-associated liver diseases has been well studied and is extensively reviewed here. Several studies show that betaine protects against the development of alcohol-induced hepatic steatosis, apoptosis, and accumulation of damaged proteins. Additionally, it can significantly prevent/attenuate progressive liver injury by preserving gut integrity and adipose function. The protective effects are primarily associated with the regulation of methionine metabolism through removing homocysteine and maintaining cellular SAM:SAH ratios. Similarly, betaine prevents metabolic-associated fatty liver disease and its progression. In addition, betaine has a neuroprotective role, preserves myocardial function, and prevents pancreatic steatosis. Betaine also attenuates oxidant stress, endoplasmic reticulum stress, inflammation, and cancer development. To conclude, betaine exerts significant therapeutic and biological effects that are potentially beneficial for alleviating a diverse number of human diseases and conditions.

## 1. Introduction

Many medicinal herbs and food ingredients possess therapeutic properties and a few of these have been developed as functional foods [1]. Numerous scientific reports have shown that many of these natural products possess favorable biological properties that contribute to their therapeutic activities [2]. One such natural product is betaine, also known as trimethylglycine, a stable, nontoxic natural substance that is present in animals, plants, and microorganisms. While betaine was first identified in the 19th century in beets (*Beta vulgaris*) [3,4], it is also found at high concentrations in other food sources including wheat bran, wheat germ, and spinach, as well as in microorganisms and aquatic invertebrates [3]. Betaine is endogenously synthesized through the metabolism of choline [3,4], or exogenously consumed through dietary intake [5]. Due to its essential biochemical functions, many microorganisms utilize betaine and have evolved different metabolic pathways for its biosynthesis and catabolism [6].

## 2. Dietary Betaine Uptake

Betaine is a short-chain, neutral, amino acid derivative. Daily betaine intake in the human diet ranges from an average of 1 g/day to a high of 2.5 g/day in individuals who consume a diet high in whole wheat and/or shellfish [7]. Dietary betaine is thought to be absorbed across the enterocytes primarily through the sodium-dependent amino acid transport system A, however sodium-independent transport also occurs [5]. The membrane-bound betaine/γ-aminobutyric acid transporter 1 (BGT-1) is also involved in the uptake of betaine following hypertonic stress [4,5,8]. Betaine consumed either from food sources or as dietary supplements presents similar bioavailability and is catabolized to dimethylglycine and finally to sarcosine in mitochondria of liver and kidney cells [4,9].

## 3. Important Roles of Betaine

**Figure 1:**
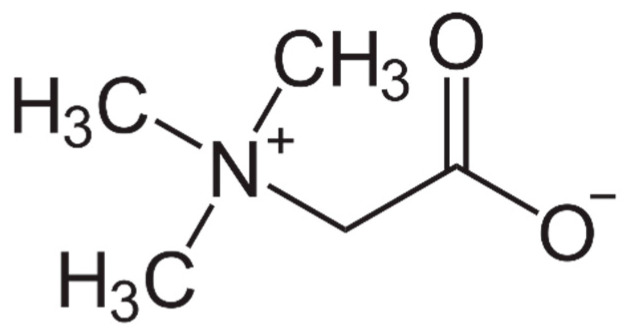
Structure of betaine.

Betaine mainly functions as (i) an osmolyte and (ii) a methyl-group donor. Because it possesses N^+^ and COO^−^ in its molecular structure, as shown in (Figure 1), betaine helps to maintain the intracellular osmotic pressure similar to other electrolytes. By exhibiting little or no binding to protein surfaces, betaine allows cells to control the surface tension of water, stabilizing both protein structure and function [4,5,8,10]. It thereby protects cells, proteins, and enzymes subjected to osmotic stress. This is particularly relevant in the kidney due to its high concentration of electrolytes and urea [11]. Betaine is the most effective osmolyte studied for the hydration of albumin [12], forming almost a complete monolayer of water around the protein and it can also maintain hemoglobin solvation [13]. In the Kupffer cells, the resident macrophages of the liver, betaine serves as an osmolyte and prevents the hyperosmolarity-induced (i) suppression of tumor necrosis factor α release and (ii) induction of prostaglandin formation and cyclooxygenase 2 expression, thereby modulating its immune function [14]. The mRNA encoding for the betaine transporter are significantly upregulated by hyperosmolarity [14]. Betaine also has an important regulatory role of organic osmolytes on human RBC membrane ATPases and it protects against hypoosmotic stress [15]. Further, by protecting skeletal muscle myosin ATPase, betaine prevents myosin structural changes due to urea [16]. Additionally, betaine affects the movement of water across the intestinal epithelium and has a role in the osmoregulation of the small intestine of broiler chicks [17,18]. In particular, betaine prevents coccidiosis (parasitic infection), an expensive disease with an estimated cost to the world’s poultry industry of USD 3.2 billion per year [19,20]. Coccidial infection disrupts osmotic balance in cells of the alimentary tract causing ionic imbalances, defective absorption, and dehydration in infected poultry These defects were reversed, and food utilization was restored after dietary betaine intake, indicating its importance in regulating colonic fluid balance and transport across the intestinal epithelium [21].

The other major function of betaine is that it donates its methyl group to the toxic metabolite, homocysteine, converting it to methionine. This reaction in catalyzed by betaine-homocysteine methyltransferase (BHMT), an enzyme that was first thought to be present primarily in the liver and kidneys [4]. Recent studies from our laboratory revealed that BHMT is also expressed in other important organs such as white adipose tissue and the intestine [22,23]. These findings have prompted additional research to evaluate betaine’s role in disease prevention [4] as well as human wellness [24].

## 4. Disease Prevention by Betaine Administration

Hepatic steatosis, defined as accumulation of excess fat in the liver [25], occurs when the mechanisms that normally utilize or remove lipids become impaired. Steatosis is the earliest manifestation of alcohol misuse or high caloric intake, but it can also be caused by insulin resistance, malnutrition, anorexia, sleep apnea, or exposure to toxins/drugs such as carbon tetrachloride, diphtheria toxins, aspirin, or tetracyclines [26]. Prolonged hepatic fat storage eventually results in metabolic dysfunction, inflammation, and advanced forms of liver disease [27,28,29,30]. The progression of liver disease from simple steatosis to hepatitis to cirrhosis and hepatocellular carcinoma is correlated with many factors, including excessive alcohol consumption, bacterial/viral infections, high body mass index, fat distribution, diabetes, race, ethnicity, genetics, and gender [31,32,33].

Alcohol-related and metabolism-associated fatty liver disease (ALD and MAFLD, respectively) are the most common causes of chronic liver disease worldwide [25,34,35]. The World Health Organization estimates that 2.3 billion people actively consumed alcohol in 2018 [36]. Excessive alcohol consumption is the third-leading preventable cause of death in the United States [37,38]. It is the most common cause of end-stage liver disease with 50% of cirrhosis-related mortality attributed either directly or indirectly to excessive alcohol use [39]. In particular, alcoholic hepatitis, a severe syndrome of ALD that is characterized by rapid onset of jaundice, malaise, tender hepatomegaly, and subtle features of systemic inflammatory response, represents a significant public health burden with almost 0.1% of all admissions related to this condition [40].

MAFLD, previously known as non-alcoholic fatty liver disease (NAFLD), is an umbrella term for liver disease unrelated to alcohol consumption and is most commonly associated with metabolic syndrome. MAFLD is characterized primarily by storage of excess macrovesicular fat due to an imbalance between the homeostatic mechanisms that regulate synthesis versus utilization of fat in liver cells [41] and is histologically indistinguishable from alcohol-induced hepatic steatosis. Studies show diabetes is a major risk factor for MAFLD [42]. The progression of MAFLD to metabolic-associated steatohepatitis (MASH), characterized by inflammatory changes in the liver, is accompanied by an increase in all-cause mortality as well as liver-related mortality. MASH is rapidly emerging as a leading cause of liver transplantation worldwide [43]. Diverse experimental animal models were examined to establish the characteristics and pathogenesis of ALD and MAFLD initiation and progression (Table 1), thus providing significant clues to the critical molecular targets to treat these fatty liver diseases [4,27,28,44,45]. Studies have focused on betaine as a treatment due to its classification as a lipotrope, i.e., an agent that reduces or prevents the accumulation of fat in the liver [46,47]. In this review, we summarize the role of betaine in restoring normal hepatic function in diseases of diverse etiologies, with special references to ALD and MAFLD.

### 4.1. ALD

Alcohol globally accounts for 3.3 million deaths each year [39,48,49,50,51,52]. An estimated 2.4 billion individuals worldwide consume alcoholic beverages [39] in social settings without experiencing harmful effects [53,54]. However, harmful use of alcohol is responsible for 5.1% of the global burden of disease and long-term excessive consumption is inextricably linked to liver disease [55], resulting in a costly socioeconomic and medical burden [54]. The liver is the major target organ with the greatest degree of tissue injury from excessive drinking because it is the primary site of ethanol metabolism [33,56]. Alcohol metabolism occurs by three distinct pathways. In the first major pathway, alcohol is oxidized to acetaldehyde via alcohol dehydrogenase (ADH), a NAD^+^-requiring enzyme expressed at high levels in hepatocytes. The second major pathway involved in alcohol oxidization is the microsomal ethanol oxidizing system (MEOS), largely catalyzed by cytochrome P450-2E1 (CYP2E1). In the third pathway, alcohol oxidation is catalyzed by catalase in peroxisomes [57]. Acetaldehyde, generated by these three pathways, then enters the mitochondrion and is oxidized to acetate by aldehyde dehydrogenase (ALDH)-mediated catalysis [58,59].

#### 4.1.1. Stages of ALD

ALD is a disease spectrum, consisting of three major stages: steatosis or fatty liver, steatohepatitis, and fibrosis and/or cirrhosis [31]. Steatosis is characterized by intrahepatic deposition of mostly triglycerides and cholesterol esters in the form of lipid droplets [60,61,62]. While initially in perivenular hepatocytes that surround the central vein of the liver lobule, steatosis progresses to mid-lobular hepatocytes, and then extends to the periportal hepatocytes that surround the hepatic portal vein [63,64,65]. Steatosis is a reversible state that can resolve upon cessation of alcohol consumption [66]. If left untreated, steatosis can advance to steatohepatitis, characterized by inflammation and neutrophil infiltration [67]. An additional pathological feature observed in the steatohepatitis stage is “ballooning” hepatocytes, which appear swollen and reveal cellular damage [31,68,69]. The activation of Kupffer cells and their subsequent loss, the degeneration of sinusoidal endothelial cell fenestrations, and the infiltration of circulating macrophages and neutrophils, define the inflammatory changes [70,71] and subsequent hepatocyte damage observed during the development of steatohepatitis. This progressive injury, in-turn, activates hepatic stellate cells (HSCs) which are key players in the development of fibrosis [72,73]. Activated HSCs proliferate and become the principal source for the increased and irregular deposition of extracellular matrix components which replace the normal matrix with dense basement-membrane-like collagen, characteristic of fibrosis [74,75,76,77]. Furthermore, HSCs accelerate inflammatory cytokine production, drawing even more inflammatory cells and amplifying hepatocyte damage [77,78] and fibrotic changes that alter hepatic lobular organization, characteristic of hepatic cirrhosis [71].

Several mechanisms have been proposed for the development and progression of ALD including acetaldehyde toxicity, oxidative stress, increased intestinal permeability-induced endotoxemia, Kupffer cell activation, production of cytokines and chemokines, a compromised immune system, nutritional deficiencies, and altered methionine metabolism [33,52]. Previous reports from many laboratories, including ours, have demonstrated that ethanol consumption impairs several of the steps in methionine metabolism [23,60,79,80,81,82,83]. Methionine is an essential amino acid that is not only needed to initiate protein synthesis but is equally important for generating the universal methyl-group donor, S-adenosylmethionine (SAM). Chronic alcohol consumption decreases SAM levels in the liver [23,84,85] and elevates both homocysteine [83,86] and S-adenosylhomocysteine (SAH) levels [23,87,88] to ultimately decrease the hepatic SAM:SAH ratio [23]. These alterations primarily occur because of the ethanol-induced inhibition of methionine synthase (MS) which is involved in removing SAH by remethylating homocysteine to generate SAM [23,60,81,82]. The consequence of the reduced SAM:SAH ratio is impaired function of several crucial hepatic methylation reactions catalyzed by specific methyltransferases [89]. Their impaired function ultimately results in the generation of hallmark features of ALD, including steatosis, apoptosis, accumulation of damaged protein, and proteasome inhibition [23,33,60,81,82,90,91,92,93].

#### 4.1.2. Betaine Protects against the Development of Alcohol-Induced Hepatic Steatosis

Alcohol-induced fat accumulation in the liver is caused by increased uptake of adipose derived free fatty acids, accelerated de novo lipogenesis, decelerated mitochondrial fatty acid oxidation, and reduced very low-density lipoprotein (VLDL) export [94,95,96]. These alterations result from the alcohol-induced change in activities of several enzymes, transcription factors, and signaling events.

Phosphatidylethanolamine N-methyltransferase (PEMT) is an important liver enzyme that catalyzes the three successive methylations of phosphatidylethanolamine (PE) to form phosphatidylcholine (PC) [97]. The PC species generated by this pathway is an essential constituent of VLDL and hence impairment in PEMT-catalyzed PC generation reduces VLDL synthesis and secretion to retain lipids in the hepatocytes, causing their accumulation [97,98,99,100]. The transcription factor sterol regulatory element-binding protein (SREBP) regulates lipid synthesis in liver and other tissues [101]. Peroxisome proliferator-activated receptor-α (PPARα) belongs to the nuclear hormone receptor superfamily and, in the liver, functions as a lipid sensor to regulate the genes that encode the enzymes for oxidation, transport, and export of free fatty acids [102].

Alcohol consumption enhances the fatty acid synthesis proteins and the levels of SREBP and fatty acid synthase (FAS) in the liver [103,104]. In contrast, alcohol downregulates the lipid metabolism regulatory proteins, PPARα, AMP-dependent protein kinase (AMPK), and adiponectin receptor-mediated signaling, which play significant roles in lipid homeostasis [95,104]. Studies have also shown that PPARα activity/expression is downregulated in livers of ethanol-fed mice [105,106]. In addition, alcohol treatment inhibits AMPK activity, which occurs via increased intrahepatic-ceramide-levels-induced protein phosphatase 2A (PP2A) activation in mice and cultured hepatoma cells [107,108]. Zhang et al., however, reported that alcohol-induced reduction in PP2A methylation promotes the phosphorylation of forkhead box O1 (FOXO1), ultimately leading to triglyceride accumulation in the liver [109]. Long-term alcohol exposure also diminishes mitochondrial oxidative phosphorylation, which promotes hepatocyte damage by decreasing respiratory efficiency and promoting oxidant stress [110,111]. Further, alcohol administration has also shown to impair PEMT activity, resulting in reduction in VLDL secretion [90,112]. Studies conducted in several rodent models have also characterized how alcohol-induced changes in the adipose–liver axis promote hepatic steatosis. One of the most well-documented phenomena is the reduced secretion of the adipokine and adiponectin, and the impaired expression of hepatic adiponectin receptors, contributing to the development of alcohol-induced liver steatosis [113].

**Figure 2:**
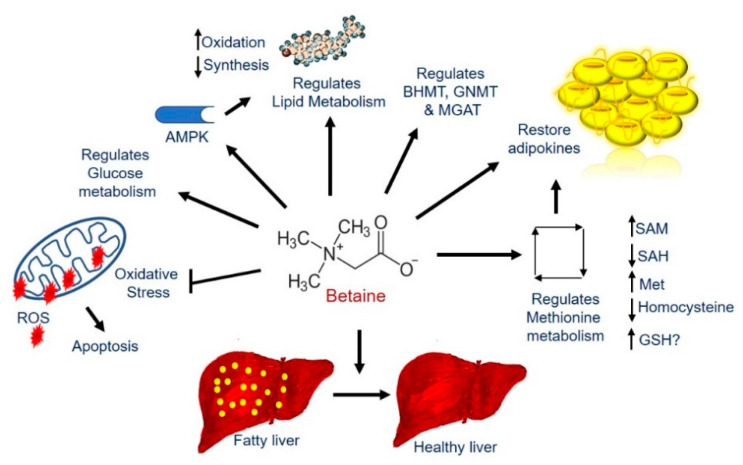
Schematic representation for the functions of betaine in liver.

Interestingly, all the above-mentioned events that promote hepatic fat accumulation are (i) indirectly or directly related to alterations in methionine metabolism and (ii) mitigated by betaine treatment as shown in multiple investigations [23,60,61,81,82,83,90,91,114,115,116], as schematically represented in Figure 2. Betaine treatment exerts protection against ethanol-induced injury by restoring the intrahepatic SAM:SAH ratio [23] and maintaining normal methylation activity [60,81,82] by providing a methyl group to homocysteine. This reaction is catalyzed by an alternate enzyme, BHMT, that, like MS, remethylates homocysteine [86] to remove SAH [23,88] and generate methionine necessary for SAM synthesis [23,117,118,119,120], and thereby maintain the hepatic SAM:SAH ratio [23]. Consequently, the activity of PEMT is preserved, leading to normal levels of VLDL secretion [90]. In addition, betaine prevents/attenuates alcohol-induced hepatic steatosis by restoring FOXO1 transcriptional activity via methylating and activating PP2A [109] and suppressing the synthesis of the rate-limiting enzyme in triglyceride synthesis, diacylglycerol acyltransferase 2 [121,122]. Song et al. reported that betaine restores the serum adiponectin levels in ethanol-fed rats by increasing its production in adipose tissue [123]. Betaine administration activates AMPK, which enhances genes encoding proteins involved in fatty acid transport and fatty acid oxidation, while decreasing fatty acid synthesis [124,125], thereby preventing triglyceride and cholesterol accumulation in the liver [115]. Betaine treatment also blocks alcohol-induced nitric oxide synthase 2 (NOS2) and nitric oxide generation, which preserves mitochondrial function [61]. Thus, betaine administration protects against the development of alcohol-induced liver injury by restoring methylation potential, increasing mitochondrial oxidation, and decreasing both the uptake of adipose derived free fatty acid and de novo lipogenesis.

#### 4.1.3. Betaine Prevents Other Indices of Early Alcohol-Induced Liver Damage

Our laboratory has also demonstrated that alcohol-induced alterations in the rat hepatocellular SAM:SAH ratio and the resulting impairment in the activities of isoprenyl carboxyl methyltransferase (ICMT), L-isoaspartyl methyltransferase (PIMT), and protein arginine N- methyltransferases (PRMT), which, respectively, regulate apoptosis [91], cause accumulation of damaged proteins [92] and inhibit proteasome activities [126]. Betaine, by restoring the methylation potential and normalizing the activities of the three methyltransferases, mitigates or eliminates these defects [23,81,82,90,91,92].

#### 4.1.4. Betaine Prevents Oxidative Stress and Inflammation in ALD

Oxidative metabolism primarily occurs in the mitochondrion, where reactive oxygen species (ROS) are generated as byproducts of biological energy-generating reactions. The body has antioxidant enzymes and antioxidants that comprise the detoxification system to remove or neutralize ROS and free radicals under normal physiological conditions [127,128]. Increased ROS generation that surpasses the capacity of the detoxification systems alters the stability of nucleic acids, proteins, and the lipid membranes of cells, compromising cellular function and promoting inflammation [129]. Alcohol consumption induces NADPH oxidase in Kupffer cells, which, by generating greater quantities of ROS causes activation of the transcription factor, nuclear factor-κB (NF-kB), that enhances tumor necrosis factor alpha (TNF-α) production to promote liver damage [130]. The MEOS pathway is also induced by alcohol consumption [131] that generates higher quantities of ROS causing oxidant stress and progressive hepatocyte injury [132]. Betaine is anti-inflammatory by its ability to upregulate antioxidant defense system [133]. Ethanol-induced increases in several factors involved in the development of inflammation, such as cluster of differentiation 14 (CD14), TNFα, cyclooxygenase-2 (COX2), growth arrest and DNA-damage-inducible 45β (GADD45β), LPS-induced TN factor (LITAF), janus kinase 3 (JAK3), toll-like receptor 2 (TLR2), toll-like receptor 4 (TLR4), interleukin 1β (IL1β), programmed cell death 4 (PDCD4), and NOS2, are all suppressed by betaine supplementation [115,133,134,135,136]. In addition, betaine supplementation also prevents alcohol-induced depletion of hepatic cysteine and glutathione (GSH). Altogether, betaine supplementation improves oxyradical scavenging activity in liver tissues altered by chronic alcohol consumption [133]. Treatment with betaine reduces the alcohol-induced elevations in serum ALT and AST [115]. Importantly, betaine administration also prevents the blood-alcohol-level cycle and significantly reduces the blood alcohol level by promoting the phenylethanolamine N-methyltransferase-mediated conversion of norepinephrine to epinephrine and increasing the metabolic rate [135].

Alcohol induces epigenetic modifications such as histone modifications (acetylation/phosphorylation/methylation/ubiquitylation/sumoylation), methylation status of DNA (hypomethylation/hypermethylation), and changes in miRNAs [137]. Epigenetic regulation of genes relevant to ALD disease pathogenesis is closely related to the underlying ethanol-induced reduction in the hepatic SAM:SAH ratio and reduced gene body methylation in all autosomes and in specific gene body sites in NOS, each of which were prevented by betaine administration [134]. Betaine prevented the formation of Mallory–Denk bodies through epigenetically attenuating the decrease of methionine adenosyltransferase 1A (MAT1A), S-adenosylhomocysteine hydrolase (SAHH), BHMT, and adenosylmethionine decarboxylase 1 (AMD1) expression and inhibiting the increase of methylenetetrahydrofolate reductase expression [138].

#### 4.1.5. Betaine Protects against the Detrimental Effects of HCV and Ethanol on Innate Immunity

Interferon type 1 (IFN type 1) response is crucial for protection of the host from many viruses including hepatitis C virus (HCV). HCV is a hepatotropic virus [139]. The progression of hepatitis is regulated by susceptibility of hepatocytes to viral infections that depends on activation of innate immunity, namely transduction of the IFN signal to activate anti-viral genes. IFN type I binds to the receptors on the cell surface of hepatocytes to induce signal transducer and activator of transcription (STAT1) and STAT2 phosphorylation followed by IRF9 (an additional factor) and their attachment to the interferon-stimulated response element (ISRE) area of DNA to activate interferon-stimulated genes (ISGs). There are certain inhibitors that block IFN signaling. One of them is protein inhibitor of activated STAT 1 (PIAS1), which attaches to STAT1 when STAT1 is not methylated by PRMT1.

**Figure 3:**
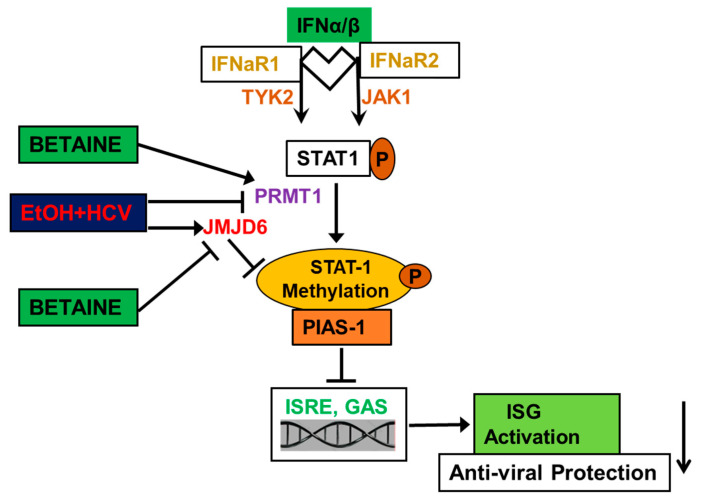
Schematic representation for effects of betaine on HCV and ethanol-mediated innate immunity.

In our studies, we found that IFN response in hepatocytes is suppressed by HCV, and this effect is potentiated by ethanol metabolism [140,141,142,143]. As schematically shown (Figure 3), acetaldehyde interferes with the attachment of STAT1 to DNA due to complex formation between phosphorylated/non-methylated STAT1 and an inhibitor of IFN signaling, PIAS1. This is attributed to impaired arginine and lysine methylation of STAT1 by PRMT1, which allows STAT1 to bind PIAS1, thereby preventing activation of anti-viral ISGs. This effect was reversed by betaine [143]. Furthermore, treatment with AMI (an arginine-methylation inhibitor), BIX (a lysine-methylation inhibitor) and tubercidin (a pan-methylation inhibitor), all mimicked the effects of acetaldehyde by suppressing the attachment of STAT1 to DNA. This led to reduced activation of ISGs with anti-viral properties, such as OAS1, OASL, viperin, and protein kinase R, which were restored by betaine co-treatment [143]. In addition to impaired PRMT1-mediated STAT1 methylation suppressing IFN signaling in hepatocytes, we also observed that ethanol metabolite induced an increase in levels of a demethylase, jumonji domain-containing 6 protein (JMJD6), generating demethylated STAT1. Betaine attenuated ethanol metabolite-induced upregulation of JMJD6, thereby increasing protective effects of anti-viral ISGs in HCV-infected hepatocytes [141]. Thus, betaine reverses alcohol-induced suppression in STAT1 methylation by PRMT1 and decreases de-methylation of STAT1 by JMJD6, thereby restoring IFN signaling and inducing anti-viral effects via stimulation of ISGs in HCV-infected alcohol-exposed hepatocytes.

#### 4.1.6. Betaine Protects against Fulminant Liver Failure and Toxin-Induced Liver Damage

Fulminant hepatic failure is characterized as severe liver injury with impairment of synthetic capability of liver cells and encephalopathy (decline in brain function) in patients with previous normal liver or, at least, well-compensated liver disease [144]. Rasineni et al. showed that betaine could prevent fulminant liver failure induced by LPS-galactosamine in mice by attenuating the activation of caspase-3 and apoptosis [145]. The toxic effects of carbon tetrachloride (CCl4) on hepatocytes are manifested histologically as hepatic steatosis, centrilobular necrosis, and ultimately cirrhosis. Betaine supplementation to CCl4 -injected rats significantly reduced hepatic lipidosis [146] and reduced the toxic effects of CCl4 on cell organelles [147]. Betaine supplementation also alleviated CCl4-induced fibrosis by inhibiting lipid peroxidation, hepatic inflammation, and expression of transforming growth factor-β1 [148].

### 4.2. MAFLD

MAFLD is defined by macrovesicular steatosis in hepatocytes, in the absence of a secondary cause such as alcohol or drugs. It is a leading cause of chronic liver disease worldwide [149]. MAFLD progresses from simple liver steatosis to steatohepatitis, and in more severe cases, to liver fibrosis, cirrhosis, and hepatocellular carcinoma [150,151]. The cause for hepatic fat accumulation in MAFLD includes increased fat accumulation in the liver from high caloric intake with persistent adipocyte derived FFA delivery and uptake by the liver, increased de novo hepatic lipogenesis, and decreased VLDL export from hepatocytes [27,152,153]. The molecular mechanism is very similar to what was discussed before in the context of ALD pathogenesis, including upregulation of key transcription factors including SREBP-1, which enhances expression of lipogenic enzymes including FAS, acetyl-CoA carboxylase, and stearoyl-CoA desaturase [154].

Lu et al. reported that a diet deficient in one-carbon methyl groups such as betaine, choline, folate, and methionine results in the development and progression of fatty liver disease by affecting specific changes in genes involved in one-carbon metabolism [155,156]. Betaine supplementation to male mice fed a high-fat diet prevented betaine deficiency, insulin resistance, and fatty liver, and normalized serum ALT levels [27]. It has also been shown that betaine supplementation to rats fed a high-fat diet upregulates the mRNAs encoding BHMT, GNMT, and MGAT, all key enzymes of one-carbon metabolism involved in regulating fat metabolism [157]. Further, betaine supplementation decreased hepatic lipid accumulation by slowing lipogenesis and enhancing lipophagy in ApoE−/− mouse models through enhanced expression of PPARα and elevated fatty acid oxidation by upregulating expression of mitochondrial and extra-mitochondrial fatty acid oxidation enzymes [158]. In addition, betaine increased AMPK, fibroblast growth factor 10, and adipose triglyceride lipase levels while suppressing lipid-metabolism-related genes in ApoE−/− mice fed a high-fat diet [159]. Mice fed a high-sucrose diet also exhibit significant fat accumulation and increased lipogenic activity in the liver similar to what was seen in high-fat diet administration, which were attenuated with betaine treatment via upregulation of AMPK [160]. Betaine supplementation to mice with MAFLD induced by methionine- and choline-deficient diet alleviated steatosis, inflammation, apoptosis, and oxidative stress, normalized mitochondrial size and respiratory chain function, stimulated β-oxidation of fatty acids, increased the number of autophagosomes, and restored both glutathione content and antioxidant enzyme activities in livers [28,161,162,163].

It is interesting to note that betaine deficiency as seen in animal models of MAFLD has been correlated with increased disease severity, a similar trend to that seen in patients [164]. Importantly, betaine treatment decreased the grade of histological steatosis, inflammation, and fibrosis in MAFLD patients [165,166]. Migilo et al. reported that oral administration of betaine for 8 weeks to MAFLD patients reduced hepatomegaly and liver-injury-marker enzymes [167]. It is evident that the efficacy of betaine needs to be tested in better-designed clinical trials for the treatment for MAFLD as well as for ALD. Betaine is indeed an attractive model compound for alleviating fatty liver diseases [168] due to its low cost, high tolerability, high solubility, and a variety of other beneficial effects as schematically shown in Figure 2.

### 4.3. Alterations in Gut–Liver and Adipose–Liver Axes in Promoting Hepatic Damage

Crosstalk between the gut and liver plays a prominent role in the pathogenesis of ALD and MAFLD [169,170]. Alcohol consumption induces intestinal dysbiosis (microbial imbalance in the gut) and increases intestinal permeability which lead to translocation of microbes and their products into the portal circulation. These products are recognized by immune receptors on resident liver macrophages (Kupffer cells) and hepatic stellate cells (HSCs) to initiate an inflammatory cascade that triggers a fibrotic response [171,172,173]. Alcohol-induced dysbiosis also promotes steatosis development [174].

White adipose tissue (WAT) plays an important role in regulating whole-body lipid and energy homeostasis [175]. It not only acts as a reservoir for energy storage, but also as a complex, essential, and highly active metabolic and endocrine organ. WAT communicates with the liver and other tissues to control lipid distribution [176] and its dysfunction is a key feature in the pathophysiology of ALD, MAFLD, and obesity-related chronic metabolic and cardiovascular diseases [177,178,179]. Most notably, during ALD and MAFLD pathogenesis, it is the accelerated WAT lipolysis and the uptake of the mobilized free fatty acids by the liver and their esterification into triglycerides which contributes to hepatic steatosis development [180,181,182]. The latter process is regulated by altered secretion of adipokines observed in animal models or in patients with metabolic disease associated with high caloric intake or alcohol misuse [183,184,185,186,187,188,189,190]. Ethanol consumption increases WAT TNF-α expression, which, by inhibiting the release of the anti-inflammatory adipokine, adiponectin [191], impairs hepatic lipid metabolism [113]. Similarly, high-caloric-intake-induced WAT lipolysis is accompanied by necrosis and inflammation in this organ, along with aberrant secretion of adipokines which contribute to hepatic damage [192,193]. Further studies have reported that chronic alcohol consumption results in impaired methionine metabolism in adipose tissue, characterized by increased accumulation of homocysteine [123] and SAH levels and a consequent decrease in the SAM:SAH ratio [194]. This loss in the methylation potential has been shown to enhance hormone-sensitive lipase (HSL) activation to promote lipolysis in WAT [123,195].

#### 4.3.1. Betaine Maintains Intestinal Epithelial Barrier Integrity

**Figure 4:**
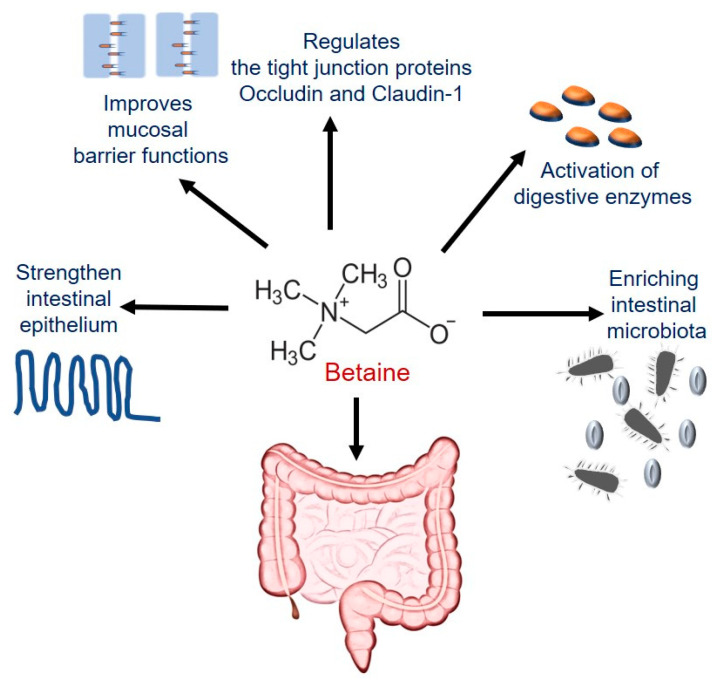
Schematic representation for the functions of betaine in the intestine.

The intestinal epithelial barrier is mainly provided by the highly specialized intercellular multiprotein junctional complex, tight junctions, located at the apical end of epithelial cells which allow minimal leakage of luminal contents into the portal circulation. If the barrier is breached, it can cause significant inflammation and, if sustained, liver damage [196]. Betaine improved the intestinal mucosal barrier by upregulating expression of zonula occludens-1 (ZO1) and occluding-tight junction proteins as well as maintained the normal gut microbiota composition in an acute liver failure model by inhibiting the TLR4/MyD88 signaling pathway [197]. Similar results were also reported on attenuation of LPS-induced decreases of the tight junction structural proteins, occluding, and claudin-1, by betaine administration to restore barrier function of porcine intestinal epithelial cells [198]. Others previously reported similar protective effects of betaine in stabilizing intestinal epithelium in coccidia-infected broiler chicks [199]. Additional beneficial effects of betaine include activation of digestive enzymes and restoring intestinal morphology and microbial diversity in high salt stressed rats [200]. Studies from our laboratory showed that betaine prevents accumulation of intracellular SAH, which is associated with intestinal barrier disruption [22]. The protective functions of betaine in the intestine are represented schematically in Figure 4.

#### 4.3.2. Betaine Maintains Adipose Function

Previous studies showed that betaine corrected abnormal adipokine (adiponectin, resistin, and leptin) levels, enhanced insulin sensitivity by improving extracellular signal-regulated protein kinase (ERK1/2) and protein kinase B, reduced endoplasmic stress, enhanced fatty acid oxidation, and restored mitochondrial function and N6-methyladenosine mRNA methylation in WAT of mice fed a high-fat diet [201,202]. Further studies showed that betaine enhanced the conversion of existing WAT to brown adipose tissue through stimulated mitochondrial biogenesis in mice fed a high-fat diet [203]. Similar protective effects of betaine have been shown in restoring alcohol-induced adipose dysfunction. Betaine restored the impaired methylation status in WAT to alleviate PP2A inhibition and prevent the persistent HSL activation and lipolysis [194] and improved circulating adiponectin levels in alcohol-fed mice [123]. Betaine treatment was also reported to reduce the hypoxia-induced expression of inflammatory adipokines, IL6, TNFα, and leptin in human adipocytes [204].

### 4.4. Protective Effects of Betaine on Other Tissues

Alcohol-induced pancreatic steatosis in rat models was effectively prevented with betaine supplementation by suppressing SREBP-1c and FAS expression [125]. Betaine also inhibited intramyocellular lipid accumulation and improved insulin resistance in mice fed a high-fat diet [203]. In addition, betaine prevented the development of isoprenaline-induced myocardial dysfunction via its antioxidant effects and by preserving mitochondrial function [205]. Further, betaine protected against cadmium nephrotoxicity by inhibiting lipid peroxidation, increasing total antioxidant status, and reducing caspase signaling cascade in renal tissues [206]. Similarly, the antioxidant properties of betaine not only prevented oxidative stress in the kidneys and liver in an experimental allergic asthma model but also improved airway inflammation of lung tissue [207].

Betaine treatment led to a substantial rise in the motor unit activity and recovery of residual power in weakened muscle tissues of patients with acute anterior poliomyelitis, which resulted in improved sense of well-being, less fatigue, and greater strength and endurance during treatment [4,7,208,209]. Singhal et al. reported that betaine via the BHMT-catalyzed pathway exerted epigenetic control and activated neuroprotective transcriptional programs in the brain of mice with multiple sclerosis by restoring the methylation potential (SAM:SAH ratio) and preventing axonal damage [210].

### 4.5. Anti-Cancer Effect of Betaine

Previous studies reported that alcohol consumption is associated with an increased risk of breast cancer development [211,212]. Hong et al. documented the anti-cancer role of betaine in alcohol-associated breast cancer cell growth and colony formation by decreasing the induction of transcription factor II-B-related factor 1 and Pol III gene transcription [213]. Further, choline supplementation (which increases circulating choline and betaine concentrations) given in conjunction with an HFD and a chemical carcinogen 7,12-dimethylbenz[a]anthracene resulted in a 55% decline in hepatocellular carcinoma tumor numbers and a 67% decrease in tumor surface area compared to non-choline-treated mice [214].

## 5. Other Beneficial Effects of Betaine

### 5.1. Effects of Betaine on General Well-Being

Chen et al. reported that circulating betaine was closely associated with better body composition and fat distribution with lower fat mass in the trunk regions of Chinese adults [215]. A recent study reported that vitamin B12 deficiency is associated with altered lipid profile and is predictive of metabolic risk [216]. Betaine administration could protect against low-vitamin-B12-induced defects given that low or no vitamin B12 elevates homocysteine levels, reduces SAM:SAH ratio and, by modulating SREBF1 and low-density lipoprotein receptor (LDLR) genes, induces cholesterol biosynthesis in human adipocytes [217].

Betaine has been shown to induce resilience to anhedonia (the inability to feel pleasure) in mice subjected to chronic social defeat stress indicating that betaine could be used as a prophylactic nutrient to prevent stress-related psychiatric disorders [218]. Decreased circulating plasma levels of betaine were also reported in patients with schizophrenia and bipolar disorder [219]. Perhaps these disorders could be treated by restoring the depleted levels given the neuroprotective role of betaine [210]. Hassanpour et al. showed decreased malondialdehyde and improved levels of both superoxide dismutase and glutathione peroxidase, all of which are characteristic of an antioxidant effect, in brains of adult mice fed a betaine-supplemented diet. They also reported betaine has an anti-nociceptive and a sedative role through interactions with opioidergic and γ-aminobutyric acid (GABA) receptors [220]. In cockerels (young roosters), betaine affected the central cholesterol metabolism by decreasing the hypothalamic content of total cholesterol and cholesterol esters and downregulating the expression of cholesterol biosynthetic genes related to brain function [221]. 

Betaine improves athletic performance as shown by the fact that its addition to a carbohydrate–electrolyte fluid-replacement beverage resulted in improved mean sprint time to exhaustion and enhanced anaerobic/aerobic metabolism [4]. A study also reports that betaine increases tolerance to hypertonic and thermal stressors at the cellular level by stimulating heat shock protein expression, reducing oxidative damage and exercise-induced gut permeability, and protecting against bacterial translocation and endotoxemia [5]. At the systemic level, chronic betaine intake lowers core temperature by reducing inflammation markers and changing blood chemistry as shown in several animal models exposed to heat stress [5,222,223]. Leng et al. stated that betaine supplementation did not affect growth performance of broilers (chickens raised for meat production), but it effectively reduced abdominal fat deposition by decreasing fatty acid synthesis and increasing β-oxidation [224]. He et al. showed significant changes in triglyceride, free fatty acid, and LDL-and HDL-cholesterol after betaine treatment of heat stressed broilers, thus improving carcass composition via modulating lipid metabolism [225]. Also, betaine supplementation in water reduced rectal temperature in broiler chickens exposed to cyclical heat stress [226] and decreased core and skin temperature of sheep exposed to the same treatment [227].

### 5.2. Effects of Maternal Betaine Supplementation on Offspring

Higher plasma betaine concentrations of pregnant women at 26–28 weeks of gestation is associated with smaller infant birth size and lower abdominal fat mass of their offspring [228]. Generally, maternal betaine supplementation normalizes fetal growth and adiposity of progeny of obese mice by reducing glucose and fatty acid transporters and the growth-promoting insulin-like growth factor 2 in the placenta [229]. Maternal betaine supplementation during gestation improved twin lamb survival and shortened time interval from birth to first suck, potentially due to increased creatine production [230]. Further, betaine supplementation to pregnant rats exposed to glucocorticoids normalized adipose lipolysis and circulating free fatty acids, and prevented ectopic lipid deposition in liver and skeletal muscle by modifying DNA methylation on the promoter sites of lipolytic genes [231]. Betaine administration decreased hepatic cholesterol deposition through epigenetic regulation of genes involved in cholesterol metabolism in juvenile chickens [232]. Betaine exerted a transgenerational effect on estrogen-responsive genes in rat offspring, which was associated with corresponding alterations in DNA methylation and the promoter of affected genes [233].

## 6. Safety Studies with Betaine

Betaine is approved for human consumption based on the effectiveness with therapeutic equivalence by the FDA under sections 505 of the Federal Food, Drug, and Cosmetic Act [236]. Oral 3 or 6 g single doses of betaine administrated in orange juice after a 12 h overnight fast to healthy human volunteers resulted in an acute and dose-dependent increase in serum betaine levels and a reduction in plasma homocysteine concentrations within 2 h [237]. Human intervention studies showed no adverse effects with 4 g/day supplemental administration of betaine in healthy subjects, however overweight subjects with metabolic syndrome showed a significant increase in total and LDL-cholesterol concentrations. These effects were not observed with 3 g/day of betaine administration [238]. Other toxicity studies reported that the LD50 in mice for betaine is 10.8 g/kg [239] when injected subcutaneously and 0.83 g/kg with intravenous injection [240]. Burnett et al. reported a similar oral betaine LD50 value of 11.1 g/kg in rats [241]. An administration of betaine to piglets at dose levels up to 20 g/kg feed for 6 weeks showed no adverse effects [238].

## 7. Conclusions and Future Perspectives

This review described the major physiological effects of betaine as a preventive agent for the treatment for various diseases (Table 1), including ALD, MAFLD, and cancer due to its properties as an osmoprotectant and a methyl-group donor. Betaine also attenuates oxidative stress, endoplasmic reticulum stress, inflammation, and cancer development. The protective effects are primarily associated with the regulation of methionine metabolism, by removing homocysteine and maintaining cellular SAM:SAH ratio. As a result, it is worthwhile to further investigate betaine because it exerts significant therapeutic and biological effects that are potentially beneficial for alleviating a diverse number of human diseases.

## Figures and Tables

**Table 1:** Protective effects of betaine in experimental animal models, cell culture systems, and clinical studies.

Therapeutic Effects of Betaine Administration	Experimental Model	Authors
Prevents hepatic fat accumulation in ALD	Male Wistar rats; C57BL/6 mice; Balb/c mice	[23,27,83,115,121,157,158,160]
Preserves/restores hepatic SAM: SAH ratios by regenerating SAM and lowering SAH and homocysteine levels in ALD	Male Wistar rats; hepatocytes; male C57BL/6 mice	[23,60,61,81,82,83,84,86,88,91,92,117,119,121,234,235]
Restores activities of various liver methyltransferases (PEMT, ICMT, PIMT, PRMT) to increase phosphatidylcholine levels, preventing apoptosis and accumulation of damaged proteins, and restoring proteasome activity	Male Wistar rats; hepatocytes	[23,90,91,92]
Suppresses the synthesis of DGAT2, a rate-limiting enzyme in triglyceride synthesis, by alleviating ERK1/2 inhibition in ALD	Male C57BL/6 mice	[121]
Upregulates antioxidant defense system and improves oxyradical scavenging activity in ALD	Male Wistar rats	[133]
Prevents/attenuates ER stress in ALD	Male C57BL/6 mice	[83]
Exerts hepatoprotection by preserving mitochondrial function in ALD	Male Wistar rats	[61]
Restores the serum adiponectin levels in ALD	Mice	[123]
Prevents elevations of CD14, TNFα, COX2, GADD45β, LITAF, JAK3, TLR2, TLR4, IL1β, and PDCD4 and NOS2 mRNA levels in alcoholic liver injury	Male Wistar rats	[115,133]
Prevents serum ALT and AST activity elevations in models of ALD and MAFLD	Male Wistar rats	[27,115,121]
Reduces liver oxidant stress, inflammation, and apoptosis in MAFLD	Male C57BL/6 mice	[28]
Remethylates homocysteine, protecting from oxidant stress and restoring phosphatidylcholine generation in MAFLD	C57BL/6 mice	[161]
Stimulates β-oxidation in livers of MCD diet-induced MAFLD	Male Sprague-Dawley rats	[162]
Alleviates steatosis and increases autophagosomes numbers in mouse livers with MAFLD	Male C57BL/6 mice; rats	[120,161]
Enhances the conversion of existing WAT to brown adipose tissue through stimulating mitochondrial biogenesis in MAFLD	Mice	[203]
Alleviates ROS-induced mitochondrial respiratory chain dysfunction in MAFLD	Male Sprague-Dawley rats	[163].
Attenuates different grades of steatosis, inflammation, and fibrosis in MAFLD patients	Human trials	[45,165,166,167]
Prevents adipose tissue dysfunction in ALD	Male C57BL/6 mice	[194]
Reduces the inflammatory adipokines, IL6, TNFα, and leptin in human adipocytes	Human visceral adipocytes	[204]
Inhibits lipid peroxidation, hepatic inflammation, and expression of transforming growth factor-β1 in liver fibrosis	Male chicks	[148]
Suppresses alcoholic liver fibrosis	Rats	[116]
Prevents the formation of Mallory–Denk bodies through epigenetic means by attenuating the decrease of MAT1A, SAHH, BHMT, and AMD1 expression	C3H male mice	[138]
Reverses the inhibitory effects of acetaldehyde on IFN signaling and decreases de-methylation of STAT1 by JMJD6	HCV-infected Huh7.5 CYP2E1 (+) cells and human hepatocytes	[141,143]
Enhances expression of PPARα and elevates fatty acid catabolism	Male C57BL/6 and ApoE−/− mice	[158].
Inhibits lipogenic activity in liver by activation of AMPK	ApoE−/− mice; Male C57BL/6 mice	[159,160]
Regulates colonic fluid balance	Rats	[21,200]
Improves intestinal barrier function and maintains the gut microbiota	Porcine epithelial cells; Caco-2 cells; rat small intestinal cell line IEC-18	[22,197,198]
Activates GI digestive enzymes and ameliorates intestinal morphology and microbiota dysbiosis	Male Sprague Dawley rats	[200]
Attenuates alcoholic-induced pancreatic steatosis	Male Wistar rats	[125]
Associated with resilience to anhedonia and prevention of stress-related psychiatric disorders	Male C57BL/6 mice	[218]
Treats asthma-induced oxidative stress, thus improving airway function of lung tissue	BALB/C mice	[207]
Protects against cadmium nephrotoxicity	Male Wistar rats	[206]
Protects against isoprenaline-induced myocardial dysfunction	Male Wistar rats	[205]
Anti-nociceptive and sedative role via interactions with opioidergic and GABA receptors	Male albino mice	[220]
Normalizes fetal growth and reduces adiposity of progeny from obese mice	C57BL/6J mice	[229]
Anti-cancer effect in alcohol-associated breast cancer cell growth and development	Breast adenocarcinoma cell line (MCF-7)	[213]
Reduces rectal temperature in broiler chickens	Chickens	[226,227]
Improves post-natal lamb survival	Lambs	[230]

## Data Availability

Not applicable.

## Abbreviations

ADH, alcohol dehydrogenase; AHCY, adenosyl homocysteinase; ALD, alcohol-associated fatty liver disease; ALDH, aldehyde dehydrogenase; AMD1, adenosylmethionine decarboxylase 1; AMPK, AMP-dependent protein kinase; BGT-1, betaine/γ-aminobutyric acid transporter 1; BHMT, betaine-homocysteine methyltransferase; CD14, cluster of differentiation 14; COX-2, cyclooxygenase-2; CYP2E1, cytochrome P450 2E1; ERK. extracellular-regulated kinase; FAS, fatty acid synthase; GABA, gamma aminobutyric acid; GADD45β, growth arrest and DNA damage-inducible beta; GSH, glutathione; HCV, hepatitis C virus; HSL, hormone-sensitive lipase; ICMT, isoprenyl carboxyl methyltransferase; IFN, interferon; IL1β, interleukin 1β; NOS, inducible nitric oxide synthase; ISGs, interferon-stimulated genes; ISRE, interferon-stimulated response element; JAK3, janus kinase 3; JMJD6, jumonji domain-containing 6 protein; LDLR, low-density lipoprotein receptor; LITAF, LPS-induced TN factor; MAFLD, metabolism-associated fatty liver disease; MASH, metabolic-associated steatohepatitis; MAT1A, methionine adenosyltransferase 1A; MEOS, microsomal ethanol oxidizing system; MS, methionine synthase; MTHFR, methylene tetrahydrofolate reductase; NAFLD, non-alcoholic fatty liver disease; NF-kB, nuclear factor kappa B; OAS1, 2′-5′ oligoadenylate synthetase-1; PC, phosphatidylcholine; PDCD4, programmed cell death 4; PEMT, phosphatidylethanolamine N-methyltransferase; PIAS1, protein inhibitor of activated STAT 1; PIMT- L, isoaspartyl methyltransferase; ROS, reactive oxygen species; PP2A, protein phosphatase 2A; PPARα, peroxisome proliferator-activated receptor-α; PRMT1, protein methyltransferase 1; SAH, S-adenosylhomocysteine; SAM, S-adenosylmethionine; SREBP, sterol regulatory element binding protein; STAT, signal transducer and activator of transcription; TNF-α, tumor necrosis factor alpha; TLR2, toll-like receptor 2; TLR4, toll-like receptor 4; VLDL, very low-density lipoprotein; WAT, white adipose tissue.
